# Preparing Enteral Formulas for Adult Patients with Phenylketonuria: A Minor Necessity but Major Challenge—A Case Report

**DOI:** 10.3390/jcm12237452

**Published:** 2023-12-01

**Authors:** Adriana Pané, Marcos Carrasco-Serrano, Camila Milad, Pere Leyes, Pedro Juan Moreno-Lozano, Roser Ventura, José Cesar Milisenda, Francesc Josep García-García, Glòria Garrabou, Judit García-Villoria, Rosa Maria López-Galera, Antonia Ribes, Josep Maria Grau-Junyent, Maria de Talló Forga-Visa, Cristina Montserrat-Carbonell, on behalf of PKU.CAT Consortium

**Affiliations:** 1 Endocrinology and Nutrition Department, Hospital Clínic, 08036 Barcelona, Spain; 2 Adult Inborn Errors of Metabolism Unit, Hospital Clínic, 08036 Barcelona, Spainfjgarcia@recerca.clinic.cat (F.J.G.-G.); garrabou@clinic.cat (G.G.); jugarcia@clinic.cat (J.G.-V.); rmlopez@clinic.cat (R.M.L.-G.); aribes@clinic.cat (A.R.);; 3 Centro de Investigación Biomédica en Red de la Fisiopatología de la Obesidad y Nutrición (CIBEROBN), Instituto de Salud Carlos III (ISCIII), 28029 Madrid, Spain; 4 Asociación Española para el Estudio de los Errores Congénitos del Metabolismo (AECOM), 28221 Majadahonda, Spain; 5 Internal Medicine Department, Hospital Clínic, 08036 Barcelona, Spain; 6 Fundació Clínic per la Recerca Biomèdica (FCRB), Institut d’Investigacions Biomèdiques August Pi Sunyer (IDIBAPS), 08036 Barcelona, Spain; 7 Centro de Investigación Biomédica en Red de Enfermedades Raras (CIBERER), Instituto de Salud Carlos III (ISCIII), 28220 Madrid, Spain; 8 Inherited Metabolic Diseases and Muscle Disorders Laboratory, FCRB-IDIBAPS, Faculty of Medicine and Heath Sciences, University of Barcelona, 08036 Barcelona, Spain; 9 Division of Inborn Errors of Metabolism-IBC, Biochemistry and Molecular Genetics Department, Hospital Clínic, 08036 Barcelona, Spain

**Keywords:** adult phenylketonuria, dietary management, enteral nutrition, enteral tube feeding, protein substitute, clogging

## Abstract

Phenylketonuria (PKU) is the most frequent of the congenital errors of amino acid (AA) metabolism worldwide. It leads to the accumulation of the essential AA phenylalanine (Phe) and it is associated with severe neurological defects. The early diagnosis and treatment of this rare disease, achieved through newborn screening and low-Phe diet, has profoundly changed its clinical spectrum, resulting in normal cognitive development. We face the first generation of PKU patients perinatally diagnosed and treated who have reached adulthood, whose special needs must be addressed, including feeding through enteral nutrition (EN). However, recommendations regarding EN in PKU constitute a gap in the literature. Although protein substitutes for patients with PKU are offered in multiple forms (Phe-free L-amino acid or casein glycomacropeptide supplements), none of these commercial formulas ensures the whole provision of daily total energy and protein requirements, including a safe amount of Phe. Consequently, the combination of different products becomes necessary when artificial nutrition via tube feeding is required. Importantly, the composition of these specific formulas may result in physicochemical interactions when they are mixed with standard EN products, leading to enteral feeding tubes clogging, and also gastrointestinal concerns due to hyperosmolality. Herein, we present the first reported case of EN use in an adult patient with PKU, where the separate administration of protein substitutes and the other EN products avoided physicochemical interactions.

## 1. Introduction

Phenylketonuria (PKU) is the most frequently diagnosed inborn error of amino acid (AA) metabolism. It is caused by mutations in the *phenylalanine hydroxylase* (PAH) gene that result in a reduction or elimination of the activity of the enzyme PAH, which converts phenylalanine (Phe) into tyrosine (Tyr). When PKU is diagnosed within the newborn period, early nutritional therapy prevents severe brain damage [1]. 

Thanks to the Newborn Screening (NBS) Programs, at present, we are facing the first generation of adult patients with PKU perinatally diagnosed and treated, whose special needs must be addressed, including feeding through enteral nutrition (EN). 

Currently, there are various types of marketed EN formulas that have been specifically designed to meet patients’ nutritional requirements according to their individual medical conditions. Standard EN formulas provide a balanced mix of macronutrients, vitamins, and minerals, and are based on complete protein (casein/whey protein or a mixture of animal and vegetable protein sources) [2,3], thus providing an excessive Phe content for patients with PKU. As a result, other calorie protein-free sources must be applied to ensure proper calorie intake without exceeding the patient’s estimated Phe dietary tolerance. 

Although the need to follow a very restricted low-protein diet in adulthood is still a subject of debate, early-diagnosed or early-treated PKU patients who discontinue this diet can suffer a variety of adverse neurocognitive and psychiatric outcomes [4]. On the other hand, patients with PKU who are late-diagnosed may experience behavioral improvements and better seizure control upon the introduction of dietetic treatment [5,6]. 

Inborn errors of metabolism (IEM) can be broadly categorized into acute intoxication and non-acute intoxication types based on their clinical presentation [7]. The disorders included in the first category, such as maple syrup urine disease (MSUD), organic acidemias (OA), or urea cycle disorders (UCDs), typically manifest with acute and often life-threatening symptoms triggered by the accumulation of toxic metabolites. Conversely, the non-acute intoxication ones, such as PKU, present with chronic symptoms that appear gradually over time. Nevertheless, a strictly controlled and supplemented diet is a cornerstone for their proper management. 

Enteral tube feeding (ETF) in children with IEM may be necessary both in the acute (metabolic decompensation) and chronic settings. For instance, the nutritional management of amino- and organic-acid-related defects (OA; MSUD; UCD), glycogen storage diseases, and fatty acid oxidation disorders is based on nutrient-controlled diet and caloric support. These diseases may require continuous ETF during the catabolic phase to prevent metabolic instability and/or minimize the production of toxic metabolites and to maintain a safe nutritional status. However, few reviews on ETF in the IEM scenario have been published thus far [8,9,10]. Additionally, only one of these reviews refers to the selected EN products: standard feeds adapted to lower their protein content in the setting of OA, but without any further specification [10]. 

Focusing back on PKU, although patients with PKU are not at risk of acute metabolic decompensations, they may be exposed to the multiple care settings in which EN may be needed (major surgery, traumatic brain injury, trauma, etc.) [2,3]. 

At present, protein substitutes for PKU are available in multiple forms (powder, liquid, coated granules, or tablets) and may also contain carbohydrates, lipids, vitamins, and minerals [11,12]. However, when EN is required, a major challenge arises since commercially prepared EN formulas for PKU that provide the whole necessary amount of total energy and protein requirements, including a safe amount of Phe, are not available. It should also be highlighted that PKU formulas are not based on whole protein, but on Phe-free L-amino acids, or a combination of casein glycomacropeptide (CGMP) and Phe-free L-amino acids. Therefore, the particular composition of these formulas may result in physicochemical interactions if mixed with marketed standard EN products, leading to enteral feeding tube clogging, and gastrointestinal concerns due to hyperosmolality [13,14]. 

Multiple care consensus documents and guidelines attending the dietetic management of adults with PKU do exist [11,15,16]. However, no specific recommendations regarding EN have been published to date. On the other hand, many new EN products have been created in recent decades with a variety of intended uses, and so formula preparation from modular ingredients has been progressively abandoned [2,3,17]. Nevertheless, EN in PKU constitutes a gap in the literature. 

Presented herein is the first report of a tailored EN plan for an adult male with late-diagnosed PKU complicated by dysphagia, in whom specific EN administration measures had to be adopted to solve and prevent both mechanical (tube occlusion) and digestive EN complications. 

## 2. Case Report

A child from Morocco with severe intellectual impairment was diagnosed with classical PKU at 13 years of age when his family moved to Spain. His plasma Phe level at diagnosis was 1290 μmol/L (reference values < 110 μmol/L) and the genetic analysis revealed two mutations in compound heterozygosity in the *PAH* gene (NM_000277.3): c.493G>A (p.Ala165Thr) and c.1066-11G>A (IVS10-11G>A). Thereafter, he was placed on a Phe-restricted diet, and after 6 months of sustained biochemical improvement (dried blood spot (DBS) Phe < 600 μmol/L, which is the European target value after treatment), his behavioral problems tended to ameliorate. However, in his late thirties, he developed severe dysphagia of unknown origin, which resulted in recurrent aspiration pneumonia and weight loss of up to 20.0 kg over 6 months. 

The patient was referred from his general adult hospital to our Adult Inborn Errors of Metabolism Unit as a suitable candidate for initiating a tailored EN plan. After having confirmed the absence of any medical conditions that could compromise EN, the nasogastric tube feeding was replaced with a gastrostomy given the requirement of long-term access. Before the procedure, the patient’s laboratory results were normal (including classical biomarkers of malnutrition: albumin, prealbumin, lymphocyte count, total cholesterol), apart from high Phe levels, as expected. It was not possible to calculate the median Phe concentration in the previous 12 months, a biochemical parameter of dietary control, as only one DBS sample was available: 472 μmol/L.

His height was 171.0 cm and his current weight was 81.2 kg. Therefore, according to his body mass index (BMI = 27.8 kg/m^2^), he was classified in the overweight category according to WHO definitions. It should be taken into account that before developing severe dysphagia, his body weight was 100.0 kg (BMI = 34.2 kg/m^2^; obesity class I). 

As concerns were raised about the possibility of overestimating protein requirements due to the patient’s excess weight, a more conservative approach was preferred, using his adjusted body weight (AdBW) [18] to calculate both energy and protein requirements.

To better assess protein needs, both his usual protein intake according to 5-day food records (calculations provided by the Spanish database Odimet^®^, https://www.odimet.es/, accessed on 1 May 2019), and the European PKU guideline formula [5] were considered. Averaging the patient’s current protein intake and his estimated requirements resulted in a value of 75.0 g protein to be consumed per day, whilst the Phe tolerance was set at 850 mg per day.

In order to prevent refeeding syndrome, the patient’s caloric intake was increased stepwise along with frequent electrolyte monitoring until 70% of calories was reached, with slower adjustments thereafter. 

Although providing a formula with CGMP was thought of as one of the most suitable dietetic approaches, its start had to be delayed because of supply difficulties. Therefore, an alternative EN plan was designed combining: (1) a normocaloric-normoproteic enteral formula with fiber (Fresubin Original Fibre EasyBag^®^, (Fresenius Kabi, Bad Homburg, Germany)), (2) a Phe-free L-amino acid powdered formula (Phenyl-Free 2 HP^®^, (Reckitt Benckiser Group PLC, Slough, UK)), and (3) a protein-free milk substitute (Prozero^®^, (Vitaflo a Nestlé Health Science Company, Vevey, Switzerland)). The composition of these products can be consulted in Supplementary Table S1.

The Phe-free L-amino acid powdered formula was prepared with tap water according to manufacturer instructions and delivered as a bolus before the rest of the EN plan, which was provided in a feeding bag and using the gravity method. It should be emphasized that when the Phe-free L-amino acid powdered formula and the normocaloric-normoproteic enteral product were administered together, multiple obstruction problems occurred, as the mixture tended to clog. Moreover, under this feeding plan, the patient suffered acute diarrheic episodes, which gradually resolved. A relatively high osmolality of the final mixture was considered to be one of the plausible causes of diarrhea. 

Despite having adjusted the Phe content of the EN plan to the patient’s estimated tolerance, his DBS Phe/Tyr levels fell to very low values, which may have been due to an inaccurate quantification of Phe intake. Subsequently, increasing the total amount of natural protein through the standard EN formula was sufficient to ensure that Phe levels were kept within the therapeutic range.

When the CGMP formula became available (PKU Sphere 15^®^, (Vitaflo a Nestlé Health Science Company, Vevey, Switzerland)), along with a high-energy powdered product (Duocal^®^, (Nutricia N.V., Zoetermeer, The Netherlands)), the feeding plan was adapted accordingly (Table 1).

Following the same approach as with the Phe-free L-amino acid powdered product, the CGMP-based formula was administrated as a bolus, separately from the rest of the EN mixture. 

For the patient’s water requirements, a total of 1400 mL tap water was provided: 50 mL before and after every meal, and 250 mL between meals. Furthermore, approximately 600 mL was also provided through the commercial liquid enteral formulas, and additional tap water was offered when preparing the soluble medications. 

Although the gastric feeding was initially well-tolerated, enabling the maintenance of Phe levels within the European target range for adults (120–600 μmol/L), some episodes of vomiting were reported. Reducing the enteral infusion rate with a feeding pump was sufficient to prevent their recurrence.

**Figure 1:**
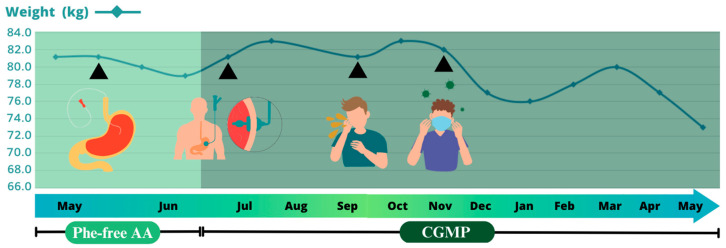
Patient’s weight evolution throughout the EN plan. The first arrowhead indicates EN start through a nasal gastric tube. The second one represents percutaneous gastrostomy placement by intervention radiology. The third arrow indicates his weight when he presented vomiting episodes due to the requirements of a higher Phe and protein intake. The last one coincides with COVID-19.

Lastly, it should be highlighted that after suffering from COVID-19 in December 2020, the patient’s caloric requirements needed to be reevaluated weekly to avoid excessive weight loss (Figure 1). 

**Figure 2:**
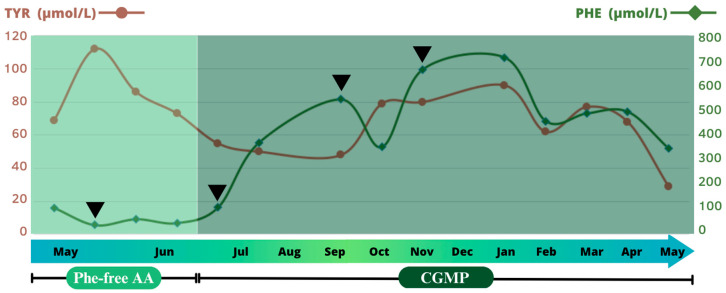
DBS Phe levels (μmol/L) during the EN plan. The first arrowhead indicates EN start through a nasal gastric tube. The second one represents percutaneous gastrostomy placement by intervention radiology. The third arrow indicates DBS Phe level increase due to vomiting episodes. The last one coincides with COVID-19.

The patient’s metabolic control based on DBS Phe levels is shown in Figure 2. Phe monitoring was performed consistently, on a monthly basis, through DBS collected in the fasting early morning hours. However, to specifically assess the impact of external factors, the frequency could be increased (to weekly or twice monthly). 

## 3. Discussion

Despite PKU being a rare condition (with a prevalence of 1/10,000 in European subjects), there is an increasing rate of PKU patients reaching adult health care thanks to their early diagnosis and treatment achieved through NBS programs. These adult patients have special needs and challenges that must be addressed, including the use of EN. Although EN represents a valuable clinical intervention in a wide range of care settings, neither the American nor European PKU guidelines provide specific recommendations to implement it under a care consensus [6,19]. Similarly, ASPEN and ESPEN guidelines do not consider patients with IEM who require EN [20,21,22]. This gap in the literature may explain some of the deficiencies identified in the knowledge and technique of EN for children with IEM in the home setting [23]. Additionally, no case reports concerning EN for adults with PKU have been published to date. Only a few cases in pediatrics, most of them focused on preterm infants or low-weight newborns, are available [24,25,26]. It should be emphasized that none of these reports consider artificial nutrition via tube feeding in exclusivity. The situation is similar to that of parenteral nutrition as only two cases have been reported thus far [27,28]. 

On the other hand, maintaining the PKU dietary plan for life is not only challenging but also constitutes a great socio-economic burden [29]. Moreover, if treatment outcomes are inadequate for long periods, patients may develop neurological impairments [30]. Before the randomized placebo-controlled trial of a Phe-restricted diet in adults with late-diagnosed PKU by P. J. Lee et al. [31], anecdotical data suggested that diet could have a positive effect on intellectual disabilities. After its publication in 2009, evidence regarding improvements in behavior due to a low-protein controlled diet became more robust. Therefore, offering PKU dietetic treatment to adults with late diagnoses appears to be advisable [5]. Nevertheless, nutritional support may become even more arduous when EN is required. 

As no study group has specifically assessed EN in the PKU setting up to now, a tailored EN plan using protein substitutes, a natural protein source, and fat and carbohydrate modules is recommended [16]. 

Currently, a wide spectrum of protein substitutes is available. To select the most suitable option, two main aspects were considered in our case. Firstly, since high-osmolality formulations may lead to delayed gastric emptying and cause osmotic diarrhea, we gave preference to CGMP preparations instead of protein substitutes based on Phe-free L-amino acids [11,13,14]. Manufacturers normally list osmolality only for a standard dilution, but enteral feedings may consist of more than one product. Knowing the final osmolality of the prepared mixtures may improve our product selection, but these data are not readily available [32]. On the other hand, the use of equations for the prediction of the osmolality of any multiproduct formula may be imprecise [13]. Even if a cause–effect correlation could not be established, some episodes of diarrhea occurred when the patient was receiving a Phe-free L-amino acid formula. Secondly, as our patient was at risk of poor bone/muscle health due to the high prevalence of vitamin D insufficiency in nursing home residents, CGMP emerged as the best option. In humans, there is evidence that CGMP improves nitrogen retention and, in animal studies, it has been proven to attenuate PKU skeletal fragility [33,34]. 

With regard to the enteral plan preparation, in order to avoid physicochemical interactions (acidic profile of AA as a risk factor for clogging if mixed with intact proteins [35], starch content as a risk factor for thickening over time [16]), both the Phe-free L-amino acid and CGMP-based formulas were always administered separately from the other enteral products through a bolus delivery [11]. Furthermore, as all protein substitutes should be prepared immediately prior to use, the efficacy of delivering PKU formulas as a bolus was reinforced. 

Another aspect that was challenging in our case was the management of the patient’s pre-obesity state. There is an assumption of higher overweight and obesity prevalence amongst adults with PKU, although this is not confirmed by recent studies [36], and therefore protein requirements should be recommended based on ideal body weight (IBW) [11]. However, since IBW is an arbitrary measurement, and as our patient had lost up to 20.0 kg, we decided to apply AdBW. The AdBW has been proposed as one method to improve the accuracy of predictive equations when calculating the caloric expenditure of patients with obesity [37]. It should be highlighted that our major concern was to not overestimate the patient’s protein requirements by using his actual body weight, whilst also bearing in mind that using IBW could result in an underestimate. Consequently, we felt more comfortable applying the AdBW as a starting point and adjusting intakes thereafter, if necessary. In order to ensure a suitable protein–energy ratio, we used the same weight for the energy calculations. 

Regarding protein requirements, the calculation is always complex. Firstly, protein needs derive from “safe levels” that are age-specific until adulthood and then remain constant over the lifespan. However, recent reviews suggest that this statement may not be appropriate for older adults with PKU, who have higher demands associated with aging [16]. Secondly, the European PKU guidelines advise an additional 20% of Phe-free L-amino acid supplements to compensate for the “digestible indispensable AA score” and an extra 20% to optimize their impact on blood Phe control [5]. But this recommendation applies to Phe-free L-amino acid supplements, and there may be differences when applied to CGMP formulas. 

Moving on to the patient’s Phe tolerance; in our case, it happened to be higher than the one that had been calculated based on his 5-day food records, or that could be expected considering his genotype [38]. Three different plausible explanations may account for this discrepancy. First, is an imprecise estimate of the patient’s food intake evaluated by regular food records. It is acknowledged that the dietary-report instruments (i.e., food frequency questionnaires, food records, 24 h dietary recalls) used in epidemiological studies are subject to reporting bias and thus might lead to measurement error, especially regarding protein intake [39,40]. Second, data on the exact Phe contents of foods are scarce and sometimes may vary between different food composition tables [41]. Third, establishing the residual activity of the PAH enzyme (a homotetramer) is complicated, as it is not a simple mean of the activity that each subunit produces by itself in vitro. It has been suggested that there may be a negative intra-allelic complementation between different mutant enzyme subunits [42].

## 4. Conclusions

In light of this case report and in the absence of care consensus documents on EN in the PKU setting, EN recommendations for the dietetic management of adults with PKU ought to be proposed. Even if severe dysphagia might result from PKU neurologic sequels, and so require the initiation of EN, other scenarios may be considered. 

The combination of PKU formulas, based on CGMP or Phe-free L-amino acid supplements and standard EN products, emerges as an option to cover both the patient’s energy and protein requirements, but may entail complications including enteral feeding tubes clogging, and also gastrointestinal concerns due to hyperosmolality. The separate administration of protein substitutes and standard EN products may solve these concerns and prevent both mechanical (tube occlusion) and digestive EN complications. 

## Figures and Tables

**Table 1:** Commercial products used for the tailored-designed EN plan.

	5:00 A.M.	10:00 A.M.	4:00 P.M.	10:00 P.M.
PKU Sphere 15^®^ powder (g) Vitaflo a Nestlé Health Science Company, Vevey, Switzerland	27.0 (1 sachet)	27.0 (1 sachet)	27.0 (1 sachet)	27.0 (1 sachet)
Fresubin^®^ Original Fiber^®^ (mL) Fresenius Kabi, Bad Homburg, Germany	150.0	200.0	150.0	-
Prozero^®^ (mL) Vitaflo a Nestlé Health Science Company, Vevey, Switzerland	150.0	200.0	200.0	300.0
Duocal^®^ (g) Nutricia N.V., Zoetermeer, The Netherlands	50.0	40.0	40.0	50.0

The compositions of the commercial formulas have been detailed in Supplementary Table S1.

## Data Availability

The data presented in this case report are available on request from the corresponding authors.

## Supplementary Materials

The following supporting information can be downloaded at: https://www.mdpi.com/article/10.3390/jcm12237452/s1, Table S1: Composition of dietary products used for the EN plan.
