# Supplementary material for: Preparing Enteral Formulas for Adult Patients with Phenylketonuria: A Minor Necessity but Major Challenge—A Case Report
Source: J Clin Med. 2023 Dec 1;12(23):7452. doi: 10.3390/jcm12237452 (PMC10707006; doi:10.3390/jcm12237452)
Supplement: Supplementary file 1 [file jcm-12-07452-s001.zip › jcm-2637740-supplementary.pdf]

Supplementary Table S1. Composition of dietary products used for the EN plan.

	Phenyl-free 2HP®	PKU sphere 15®	Fresubin Original Fibre EasyBag®	Duocal®	Prozero®
**Manufacturer**	Reckitt Benckiser Group PLC, Slouugh, United Kingdom.	Vitaflo a Nestlé Health Science Company, Vevey, Switzerland.	Fresenius Kabi, Bad Homburg, Germany.	Nutricia N.V., Zoetermeer, The Netherlands.	Vitaflo a Nestlé Health Science Company, Vevey, Switzerland.
**Description**	Vanilla flavored high-protein Phe-free L-amino acid powdered formula.	Vanilla flavored low-Phe powdered formula containing casein glycomacropeptide (CGMP).	Unflavored liquid intended for the dietary management of any patients at risk of malnutrition.	A high-energy powdered product that contains a dual energy source of carbohydrates and fat.	Protein-free, liquid blend of carbohydrate and fat.
**Energy (Kcal)**	**390.0**	**338.0**	**100.0**	**492.0**	**67.0**
**Protein equivalents (g)**	**40.0 (Phe 0.0 mg)**	**56.0 (Phe 104 mg)**<br>CGMP to AA ratio of 60:40	**3.8 (Phe 210 mg)**<br>40% casein; 10% lactoprotein; 50% soya	**0.0 (Phe 0 mg)**	**0.0 (Phe 0 mg)**
**Carbohydrates (g)**	**44.0**	**18.0**	**13.8**	**72.7**	**8.1**
• **Monosaccharides**	-	1.6	0.2	1.4	0.05
• **Maltose**	-	-	0.7	5.1	0.25
• **Sucrose**	22.0	6.3	-	-	-
• **Maltodextrin**	-	3.4	*	7.3 (maltotriose)	4.8
• **Polysaccharides (e.g. starch)**	21.3	10.4	13.8 (no starch)*	58.9	0.3
**Fibre (g)**	0.0	0.0	1.5	0.0	0.0
**Lipids (g)**	6.3	4.5	3.4	22.3	3.8
**Osmolality (mOsm/Kg H_2O)**	Dilution 1:4 = 1070 mOsm/L (osmolarity)	770	330	Dilution 1:4 = 196; 1:3 = 310; 1:2 = 525	200

The nutritional information of dietary products is expressed per 100g or 100mL. The carbohydrate profile is expressed in g/100 g or 100 mL unless otherwise specified.

*Information regarding oligo and polysaccharide content is provided as a whole. Only Fresubin Original Fibre EasyBottle® contains starch, but not the EasyBag® presentation.
